# Case Report: Two cases of PD-1 inhibitor–associated myocarditis with bidirectional ventricular tachycardia

**DOI:** 10.3389/fimmu.2026.1832047

**Published:** 2026-05-25

**Authors:** Xianglin Lian, Hongming Wang, Nuoni Wang

**Affiliations:** 1 Department of Cardiac and Pulmonary Function, Henan Provincial People’s Hospital, Zhengzhou University People’s Hospital, Zhengzhou, China; 2 Department of Oncology, Changde Hospital, Xiangya School of Medicine, Central South University (The First People’s Hospital of Changde City), Changde, China; 3 Department of Cardiac Electrophysiology, Changde Hospital, Xiangya School of Medicine, Central South University (The First People’s Hospital of Changde City), Changde, China

**Keywords:** bidirectional ventricular tachycardia, case report, immune-related adverse events, myocarditis, programmed cell death protein 1

## Abstract

**Background:**

The clinical application of targeted anti-programmed cell death protein 1 (PD-1) therapy has significantly improved the prognosis of patients with various advanced malignancies. However, life-threatening immune-related adverse events remain problematic, including rare but highly lethal myocarditis. Bidirectional ventricular tachycardia (BVT) is a rare but dangerous form of ventricular arrhythmia. If immune checkpoint inhibitor (ICI)–associated myocarditis is complicated with BVT, patients face an extremely high risk of mortality.

**Case description:**

The first case was a 45-year-old male with advanced intrahepatic cholangiocarcinoma who experienced sudden chest tightness after receiving 11 cycles of camrelizumab. Test results showed significantly elevated myocardial injury markers, and electrocardiography (ECG) indicated BVT; cardiac magnetic resonance imaging findings were consistent with acute myocarditis. A diagnosis of PD-1 inhibitor–associated myocarditis complicated with BVT was considered. Following immunotherapy discontinuation, the patient was treated with steroid therapy, antiarrhythmic drugs, and electrical cardioversion. This led to marked improvements in ICI-associated myocarditis, BVT resolution, and favorable outcomes. The second case was a 34-year-old female patient with type B1 thymoma who experienced sudden chest tightness, shortness of breath, and fatigue after receiving one second-line cycle of sintilimab. Significantly elevated myocardial injury markers were observed, and ECG findings indicated BVT. The patient was diagnosed with PD-1 inhibitor–associated myocarditis complicated with BVT. However, the patient was in critical condition. Despite receiving supportive therapies, including steroid therapy, antiarrhythmic drugs, electrical cardioversion, invasive mechanical ventilation, and extracorporeal membrane oxygenation, the patient’s condition deteriorated rapidly, ultimately leading to death.

**Conclusions:**

These two cases demonstrate that although the incidence of malignant arrhythmias such as BVT in ICI-associated myocarditis is low, the risk of sudden death remains extremely high. Therefore, we consider dynamic monitoring of myocardial injury markers and ECG in patients with ICI−associated myocarditis. Additionally, our observations suggest that steroid therapy is a crucial treatment method for patients with ICI-associated myocarditis complicated with BVT. This report adds to the growing body of evidence indicating that BVT is an important yet previously infrequently described ECG manifestation of severe cardiotoxicity associated with ICIs.

## 1 Introduction

Immune checkpoint inhibitors (ICIs) have transformed the treatment landscape for patients with advanced malignancies, delivering durable remission and long-term disease control in those with certain previously refractory tumors (1, 2). ICIs enhance the immune response against tumors by blocking malignant immune system evasion, thereby improving treatment outcomes. However, this also increases the risk of immune-related adverse events (irAEs), which are extensive in scope and affect nearly all organ systems (3, 4). Among these, cardiovascular irAEs are uncommon but often associated with severe adverse clinical outcomes and high mortality rates, thus necessitating heightened clinical vigilance (5, 6).

ICI-associated myocarditis has a high mortality rate in severe cases and typically occurs early during treatment, often within the first few weeks (7). ICI-associated myocarditis may present with a fulminant course and rapidly progressing to cardiogenic shock, life-threatening ventricular arrhythmia, or complete heart block (8, 9). However, the clinical manifestations of ICI-associated myocarditis lack specificity, and diagnosis is difficult. Currents studies indicate that electrocardiography (ECG) and myocardial injury biomarkers (e.g. troponin) should be closely monitored in high-risk patients to allow for early detection of abnormalities and facilitate further evaluation; however, the impact of proactive monitoring for ICI-related myocarditis on patient prognosis remains controversial (10, 11). In addition, evidence from prospective trials is insufficient to support the inclusion of these measures into standardized monitoring protocols (12).

ICI-associated myocarditis frequently triggers various forms of arrhythmias, especially in fulminant cases, and may be accompanied by serious adverse outcomes, such as cardiac arrest, hemodynamic instability, or death (9, 13). Although ventricular tachycardia is relatively common in clinical practice, bidirectional ventricular tachycardia (BVT) is a rare subtype. BVT is defined as a ventricular arrhythmia characterized by a beat-to-beat alternation of the QRS axis or morphology, typically shifting approximately 180 degrees in the frontal plane (14). The etiology of BVT is relatively limited, with digitalis toxicity long regarded as one of the classic causes. In ICI-associated myocarditis accompanied by arrhythmias, histopathological findings show lymphocyte and macrophage infiltration, suggesting that inflammation can contribute to the pathophysiology of arrhythmias (15, 16). However, evidence regarding specific pathological features remains limited, and existing studies have widely called for higher-quality prospective research to elucidate the causal relationships and pathological mechanisms involved (16). The definition proposed by the International Cardio-Oncology Society is currently widely adopted for diagnosing ICI-associated myocarditis; however, Deharo et al. noted that this definition is empirical and has not been systematically validated (17). Despite increasing awareness of ICI-associated cardiotoxicity, data specifically addressing ICI-associated myocarditis complicated with BVT remains scarce.

We report two cases of ICI-associated myocarditis complicated with BVT in patients with advanced solid tumors receiving programmed cell death protein 1 (PD-1) inhibitor therapy. We systematically describe their clinical features, diagnostic process, treatment strategies, and differences in prognosis, aiming to highlight that BVT represents a specific ECG manifestation of severe ICI-associated cardiotoxicity. We also emphasize the need for continuous cardiovascular monitoring and multidisciplinary collaboration during the treatment of severe ICI-associated myocarditis to improve the success rate of managing severe complications in these patients.

## 2 Case description

### 2.1 Case 1

**Figure 1:**
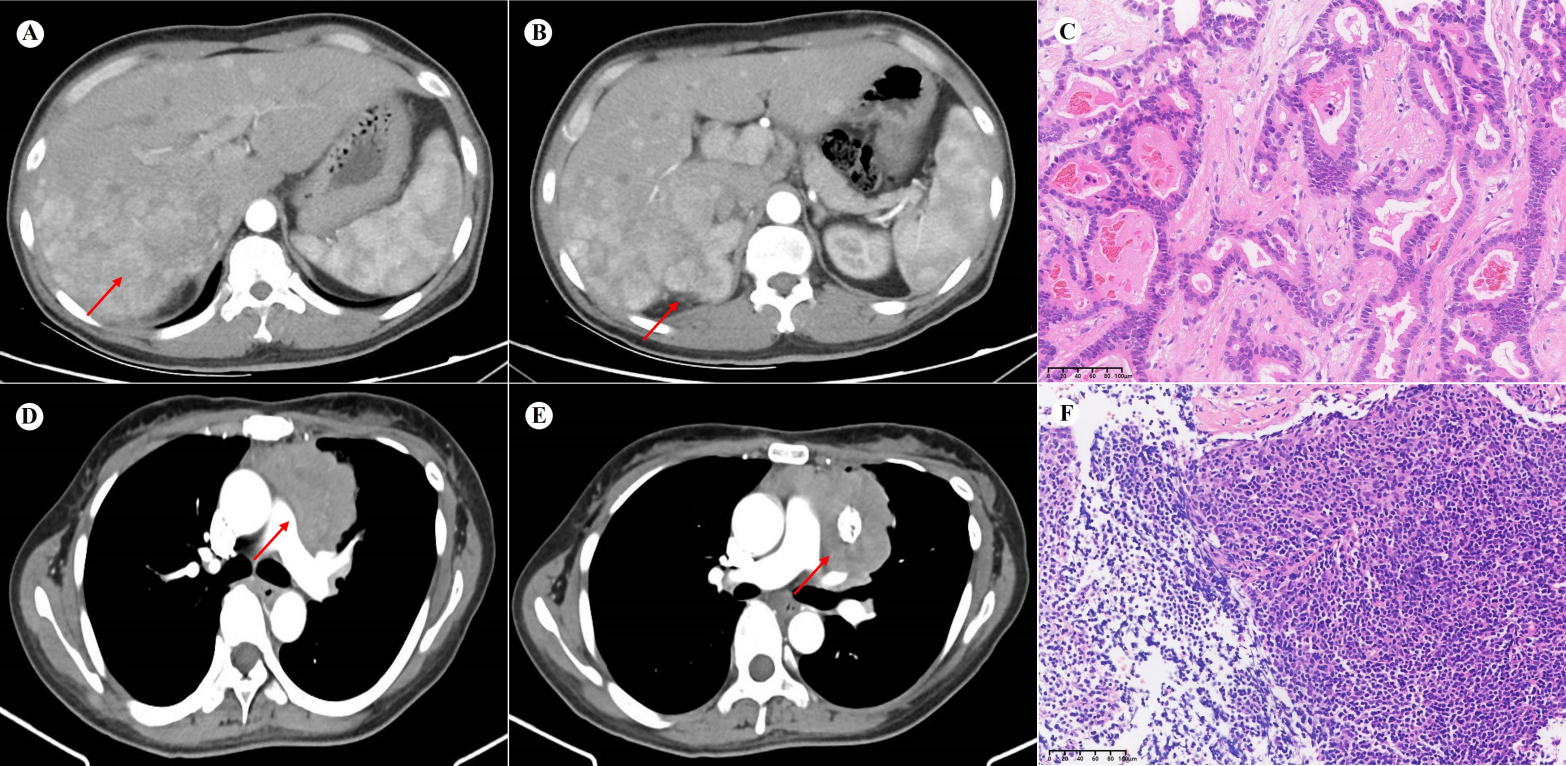
Imaging and pathological findings for the two patients. **(A–C)** Case 1. **(A, B)** the enhanced computed tomography (CT) scan performed on February 5, 2020, revealed multiple space-occupying lesions with uneven enhancement in the liver and the right adrenal gland. A percutaneous ultrasound-guided liver biopsy conducted on February 12, 2020, confirmed the diagnosis of intrahepatic cholangiocarcinoma. **(C)** hematoxylin and eosin (H&E) staining (200× magnification; scale bar = 100 µm). Immunohistochemical analysis was positive for CK7, CK19, CK8/18, SATB, Villin, and MUC-1. The Ki-67 proliferative index was approximately 10%. In contrast, stains for CK-2, CK20, Pax8, TTF-1, NapsinA, MUC-2, and MUC-4 were negative. **(D–F)** Case 2. **(D, E)** The enhanced CT scan on November 11, 2021, showed an anterior upper mediastinal mass (approximately 62 mm × 41 mm) with irregular morphology, lobulation, unclear boundaries with adjacent major vessels, and heterogeneous enhancement. A CT-guided biopsy of the mass was performed on November 16, 2021, with a postoperative pathological diagnosis of type B1 thymoma. **(F)** the corresponding H&E staining (200× magnification; scale bar = 100 µm). Immunohistochemistry was positive for CK19 (epithelial), CD3 (T cell), CD5 (T cell), CD1a, CD20 (scattered B cell), P40 (scattered), P63, CK5/6, TdT, Bcl-2 (interstitial), and MASH1 (diffuse), with a Ki-67 index of approximately 80%. Stains for CD117, EMA, desmin, collagen IV, and STAT6 were negative.

A 45-year-old male patient with no prior history of heart disease presented to the general surgery department of our hospital in February 2020 complaining of poor appetite for one month. Contrast-enhanced computed tomography (CT) revealed multiple space-occupying lesions in the liver and right adrenal gland (**Figures 1A, B**). One week later, an ultrasound-guided liver biopsy confirmed the diagnosis of primary well-differentiated intrahepatic cholangiocarcinoma with metastasis to the right adrenal gland (**Figure 1C**), with no indications for surgery. PD-L1 expression (22C3) was 3%. The patient was subsequently treated with five cycles of camrelizumab (q3w) in combination with GP (gemcitabine and cisplatin, q3w), followed by six cycles of maintenance therapy with camrelizumab (q3w) plus anlotinib (2 weeks on/1 week off). Multiple efficacy assessments during this period indicated stable disease (RECIST 1.1 criteria). Adverse reactions included grade II telangiectasia and grade I gastrointestinal reactions.

**Figure 2:**
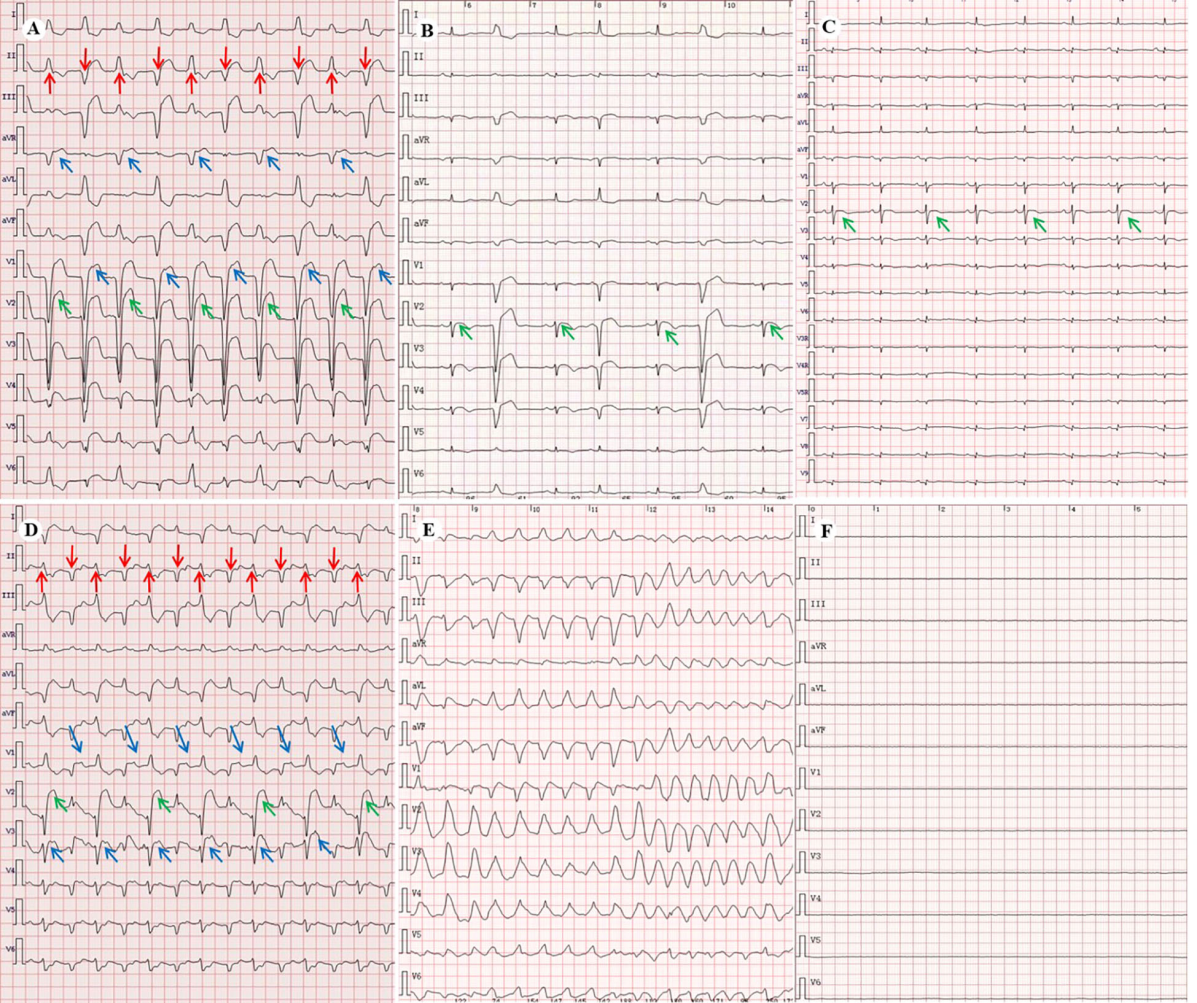
**(A–C)** Case 1. **(A)** electrocardiogram (ECG) performed on November 18, 2020, at 09:55 during the bidirectional ventricular tachycardia (BVT) episode showed a ventricular rate of 115 bpm, with generally regular RR intervals. The QRS complexes exhibited two alternating morphologies (indicated by red arrows), accompanied by 1:1 atrioventricular conduction (indicated by blue arrows), with each RS interval exceeding 100 ms. One morphology (indicated by the downward red arrow) shows precordial concordance with the dominant QRS deflection. The electrical axis alternated between left deviation and no deviation, which was particularly evident in the limb leads. Lead V1 exhibited a pattern suggestive of left bundle branch block. ECG shows a marked reverse upwardly convex ST-segment elevation (leads II, III, aVF, and V1–V4) (indicated by green arrows), strongly suggesting acute myocardial injury. **(B)** at 11:00 on November 18, 2020, following the oral administration Metoprolol and intravenous application of amiodarone, ECG showed sinus rhythm with a bigeminy of premature ventricular contractions, the morphology of which was not entirely identical to that recorded during the BVT episode. The QRS complex in the sinus rhythm exhibited upwardly convex ST-segment elevation in leads V2–V4 (indicated by green arrows), decreased QRS complex amplitude, poor R-wave progression in the precordial leads, and the formation of abnormal Q waves in the inferior leads (consistent with cardiomyocyte damage caused by myocarditis). **(C)** on January 6, 2021, following effective treatment, ECG showed gradual recovery of ST-segment elevation leads to baseline (indicated by green arrows), but with reduced QRS complex amplitude and the formation of abnormal Q waves in the inferior and right ventricular leads. **(D–F)** Case 2. **(D)** ECG performed on March 3, 2022, at 11:32 during the BVT episode showed QRS complexes exhibiting two alternating morphologies (indicated by red arrows), with regular rhythm and 1:1 atrioventricular conduction (indicated by blue arrows), with each RS interval less than 100 ms. Precordial leads showed an alternating pattern suggestive of left and right bundle branch block, with electrical alternans. ECG shows marked reverse upwardly convex ST-segment elevation in lead V2 (indicated by green arrows), similarly suggesting acute myocardial injury in the patient. **(E)** ECG performed on March 3, 2022, at 23:35 showed pulseless ventricular tachycardia-ventricular flutter, wide tachycardia complexes, followed by continuous, rapid, relatively regular large sine waves. **(F)** At 00:30 on March 4, 2022, ECG showed a flat line indicating full cardiac arrest.

**Figure 3:**
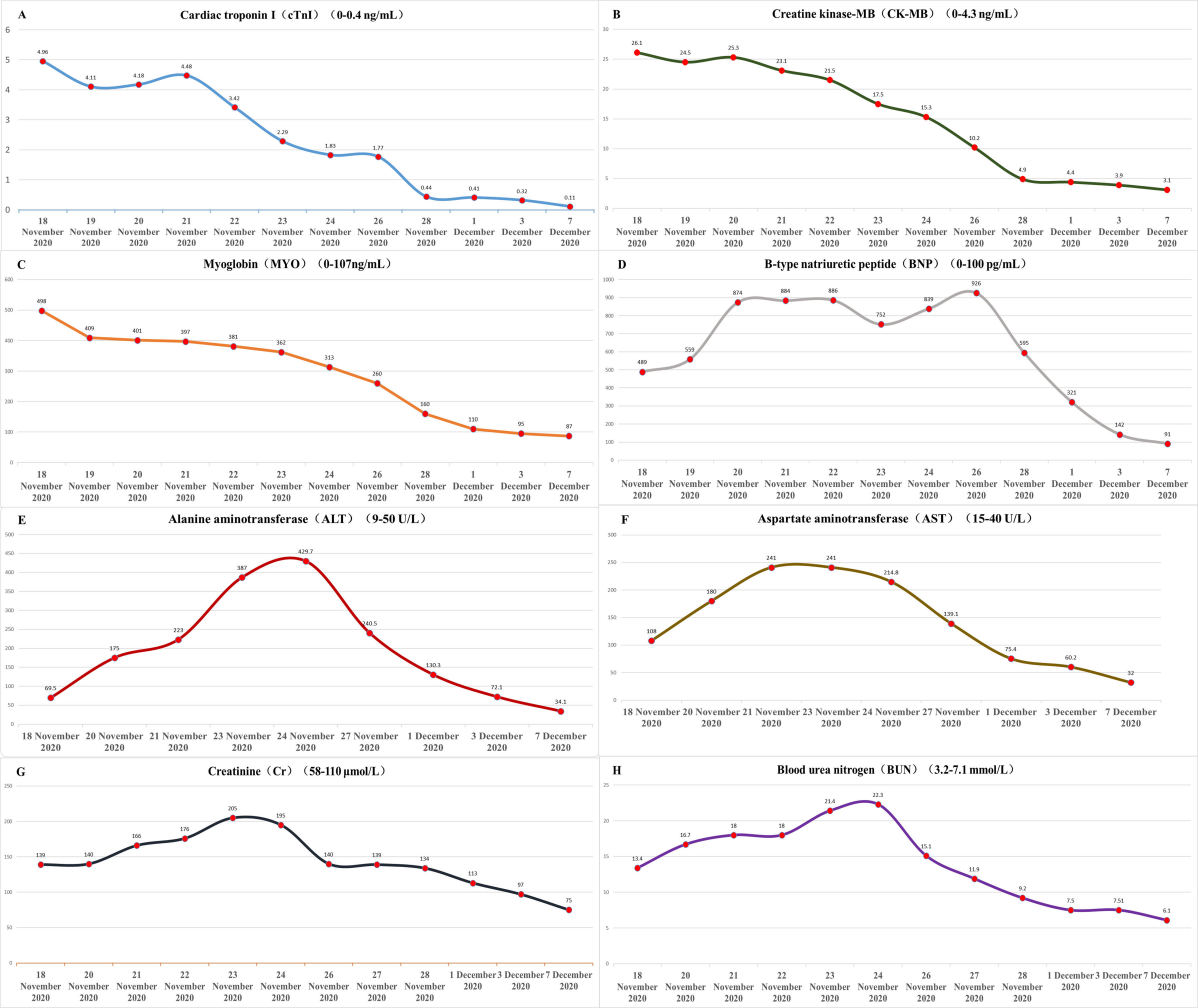
Laboratory findings during the hospital stay of the first case, from November 18, 2020, to December 7, 2020. **(A–C)** abnormal myocardial injury markers, including cardiac troponin I, creatinine kinase-MB, and myoglobin, gradually decreased following effective treatment. **(D)** B-type natriuretic peptide levels were persistently elevated and remained high for five days before gradually normalizing as the patient’s condition improved. **(E–H)** changes in liver and kidney function indicators; alanine aminotransferase, aspartate aminotransferase, blood urea nitrogen, and creatinine levels rose continuously (peaking on November 24, 2020), then steadily declined to normal ranges following steroid therapy.

In November 2020, the patient was readmitted with a sudden onset of chest tightness for one day. He did not experience limb weakness. Blood pressure was 100/60 mmHg and heart rate 115 bpm. Urgent ECG showed BVT with ST-segment elevation, indicating acute myocardial injury (**Figure 2A**). Metoprolol sustained-release tablet was administered orally as a single dose, but BVT did not resolve. Laboratory test results indicated significantly elevated myocardial injury markers (cardiac troponin I [cTnI], creatinine kinase-MB [CK-MB], and myoglobin [MYO]), cardiac dysfunction markers (B-type natriuretic peptide [BNP]), and liver and kidney function indicators (alanine aminotransferase [ALT], aspartate aminotransferase [AST], creatinine [Cr], and Blood urea nitrogen [BUN]) (**Figure 3**); however, the observed elevations in transaminases may be partly attributed to myocardial injury. After intravenous administration of amiodarone, a continuous infusion of amiodarone (20 mL/h) was initiated. Subsequent ECG monitoring indicated a transition from BVT to sinus rhythm, accompanied by frequent premature ventricular contractions in a bigeminal pattern (**Figure 2B**). The next day, bedside ultrasound echocardiography revealed localized segmental wall motion abnormalities, with a left ventricular ejection fraction (LVEF) of 59%. Over the next few days, the patient experienced recurrent episodes of ventricular tachycardia, with no indication of persistent hypoperfusion. Multiple administrations of antiarrhythmic drugs (lidocaine or amiodarone) and electrical cardioversion were performed, successfully restoring sinus rhythm. Six days after admission, follow-up assessments of relevant indicators revealed progressive deterioration in liver function (AST, ALT) and kidney function (BUN, Cr), whereas myocardial injury markers (cTnI, CK-MB, MYO) were lower than the previous results (**Figure 3**). Following multidisciplinary consultation, the patient was diagnosed with PD-1 inhibitor–associated myocarditis complicated with BVT, accompanied by acute liver and kidney dysfunction. Methylprednisolone (40 mg/day) therapy was initiated, followed by gradual tapering over three days until discontinuation. Follow-up cardiac magnetic resonance imaging (CMR) revealed findings consistent with acute myocarditis (**Supplementary Material**). In December 2020, follow-up tests showed that markers of myocardial injury and liver and kidney function had returned to normal levels. Antiarrhythmic therapy was continued during steroid treatment, with close hemodynamic monitoring. In January 2021, follow-up ECG revealed sinus rhythm (**Figure 2C**). PD-1 immunotherapy was discontinued, and regular ECG monitoring during subsequent follow-up revealed no ventricular tachycardia recurrence.

### 2.2 Case 2

A previously healthy 34-year-old female presented to the neurology department of our hospital in November 2021 complaining of lower extremity weakness and anorexia for five months. Lung contrast-enhanced CT revealed a mass in the anterior superior mediastinum, involving the pericardium and pulmonary artery (**Figures 1D, E**). A biopsy was performed a week later, with pathology suggesting type B1 thymoma (**Figure 1F**). PD-L1 expression (22C3) was 20%. The patient was found to have hyponatremia (Na+ 129 mmol/L) and hypochloremia (Cl− 89 mmol/L) on admission. Acetylcholine Receptor antibody testing was negative. Repeated nerve stimulation electromyography showed no attenuation, and the neostigmine test was negative. There were no typical myasthenia gravis symptoms, including eyelid ptosis, diplopia, or fluctuating muscle weakness. Lower extremity weakness improved after sodium supplementation; therefore, thymoma with hyponatremia was prioritized over myasthenia gravis. Due to its size and proximity to the pericardium and pulmonary artery trunk, surgical resection of the mass was not feasible. Consequently, chemotherapy was planned, and local palliative radiotherapy would be considered once the mass was smaller. The patient received two cycles of combination therapy with cisplatin, epirubicin, and endostatin. In February 2022, follow-up CT indicated progressive disease. Considering the patient’s PD−L1 expression level of 20%, the patient subsequently received one cycle of second−line immunochemotherapy with nedaplatin, albumin−bound paclitaxel, and sintilimab. Chest palliative intensity−modulated radiotherapy was initiated nine days later. A planned gross tumor volume dose of 40 Gy was prescribed to the primary lesion (2 Gy × 20 fractions). Eight fractions were delivered, for a total dose of 16 Gy.

In March 2022, the patient experienced sudden chest tightness, shortness of breath, and fatigue. Her heart rate was 140 bpm, blood pressure 90/57 mmHg, and blood oxygen saturation (SpO_2_) 99% on supplemental oxygen. Urgent ECG showed regular BVT (**Figure 2D**). Lidocaine was immediately administered via continuous infusion as antiarrhythmic therapy; beta-blockers were avoided due to the hypotension. The patient was transferred to the intensive care unit, and urgent laboratory tests revealed markedly elevated myocardial injury and cardiac dysfunction markers (cTnI 18.54 ng/mL, MYO 711.4 ng/mL, CK-MB 72.2 ng/mL, and BNP 8,406 pg/mL). The patient showed no signs of infection and had received a relatively low total radiation dose delivered to the tumor lesion located above the heart. Given the patient’s young age and low likelihood of ischemic/coronary artery disease, along with the recent history of ICI therapy, laboratory findings indicating myocardial injury, and ECG manifestations consistent with BVT, a diagnosis of ICI-associated fulminant myocarditis complicated with BVT was considered following multidisciplinary consultation. Methylprednisolone pulse therapy (500 mg) was administered. Eight and a half hours after admission, the patient suddenly lost consciousness, with undetectable blood pressure and SPO_2_ at 80%. Immediate chest compressions, bag-valve-mask ventilation, cardiac stimulants, vasopressors, fluid resuscitation, and defibrillation (for ventricular tachycardia) were initiated. Endotracheal intubation was subsequently performed for mechanical ventilation. However, the patient’s blood pressure remained difficult to maintain, and with the family’s consent, extracorporeal membrane oxygenation (ECMO) was initiated (mode: VA, blood flow: 2.0 L/min, rotational speed: 2,680 rpm, gas flow: 3.0 L/min, and FiO_2_: 100%). Three and a half hours later, bedside ECG showed ventricular tachycardia/flutter (**Figure 2E**). Despite ECMO support and high-dose vasoactive drugs, blood pressure remained difficult to maintain, and the patient’s condition continued to deteriorate. Within an hour, ECG showed complete cardiac arrest (**Figure 2F**).

**Figure 4:**
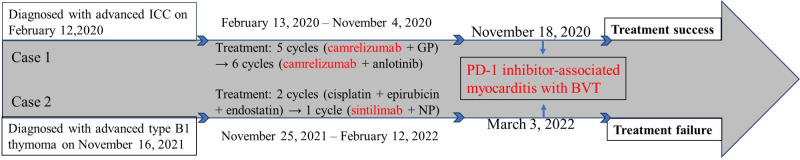
A visual overview of the therapeutic courses for the two cases. ICC, intrahepatic cholangiocarcinoma; GP, gemcitabine and cisplatin; NP, nedaplatin and albumin-bound paclitaxel; PD-1, programmed cell death protein 1; BVT, bidirectional ventricular tachycardia.

A graphical overview of the therapeutic courses for the two cases is shown in **Figure 4**.

## 3 Discussion

As ICI use becomes more widespread, the occurrence of ICI-associated myocarditis has garnered increasing attention (18). Bidirectional ventricular tachycardia (BVT) is a rare but clinically severe manifestation of immune checkpoint inhibitor (ICI)-associated myocarditis (19). Our two cases were briefly reported in a Chinese case series of five patients with BVT, which could not be retrieved via PubMed or Web of Science. Therefore, this article is intended for an international readership and provides a more detailed description and analysis from the perspective of ICI-associated myocarditis. Although BVT is classically associated with digitalis toxicity and catecholaminergic polymorphic ventricular tachycardia (CPVT), it has also been described in other inflammatory heart diseases, including fulminant myocarditis, cardiac sarcoidosis, acute myocardial ischemia, and Andersen-Tawil Syndrome (ATS). Their triggers primarily include drug (digoxin) toxicity, exercise and emotional stress, viral infection, granulomatous inflammation, and myocardial ischemia, and the corresponding clinical manifestations also show certain differences (20). Our cases therefore suggest that BVT may represent an inflammation-related arrhythmic phenotype in ICI-associated myocarditis. Notably, the arrhythmia in case 1 improved after steroid therapy and amiodarone but responded poorly to β-blockers alone, suggesting that the underlying mechanism and management in ICI-associated myocarditis may differ from those in CPVT.

Endomyocardial biopsy remains the gold standard for diagnosing myocarditis; however, its clinical application is often limited due to its invasive nature, potential risk of complications, and the presence of acute-phase hemodynamic or electrophysiological instability in some patients (21, 22). Current guidelines recommend that in cases of suspected ICI-associated myocarditis, a clinical diagnosis (or presumptive diagnosis) can be established based on clinical presentation supplemented with myocardial biomarkers (primarily troponin) and imaging modalities (primarily CMR), after excluding other etiologies and analyzing the temporal relationship with ICI use (6, 17).

The electrophysiological mechanisms underlying BVT are still poorly understood, but its core feature involves the presence of two morphologically distinct excitation foci or conduction circuits within the ventricles that are activated on a beat-by-beat basis. The primary mechanisms include: (i) alternating “dual exits” in the conduction system, wherein the ectopic focus resides in the His-Purkinje system, with electrical signals alternately transmitted due to the differential refractory periods of the left anterior/posterior branches (23); (ii) calcium overload inducing delayed afterdepolarization, causing alternating discharge from the excitation points at the two sites (24); (iii) involvement of a reentry circuit, a common pathway connecting two exits with differing refractory periods, forming a macro-reentrant circuit, or a premature beat activating a second focus via reentry (25); and (iv) dual parasystolic foci, where two independent ectopic foci, protected by an entrance block, alternately dominate ventricular excitation (23, 26). In this report, both patients developed BVT in the setting of acute or fulminant myocarditis. Neither had received digitalis, had evidence of viral infection, nor had a history of structural heart disease. Serum calcium levels at the onset of BVT were within the normal range (2.18 mmol/L and 2.12 mmol/L). Given that BVT is primarily driven by increased intracellular rather than serum calcium, we speculate that inflammation in ICI−associated myocarditis may enhance ryanodine receptor sensitivity, promoting spontaneous sarcoplasmic reticulum calcium release. This, in turn, could lead to intracellular calcium overload and delayed afterdepolarizations (DADs), with bi−focal triggered activity within the His–Purkinje system ultimately giving rise to BVT (19). In this case report, neither patient underwent genetic testing for catecholaminergic polymorphic ventricular tachycardia (CPVT) because such testing was not available at our institution at the time. As a result, molecular evidence for CPVT is lacking, which represents a limitation of this study.

In rare cases, ICI-associated myocarditis can present as wide QRS tachycardia or BVT-like, and this is typically accompanied by significant left ventricular dysfunction. However, few case reports have documented the occurrence of BVT or BVT-like wide QRS tachycardia in ICI-associated myocarditis. Alhumaid et al. reported a case of ICI-associated myocarditis with slow BVT in a patient receiving pembrolizumab; after ICI discontinuation and plasmapheresis, the myocarditis and arrhythmia resolved (19). Similarly, Tang et al. reported a case of wide QRS tachycardia resembling BVT after treatment with sintilimab, which was ultimately diagnosed as ICI-associated myocarditis (27). In differential diagnosis, other causes such as digitalis toxicity and ischemia should be excluded, and early initiation of immunotherapy (e.g., glucocorticoids and immunoglobulin therapy) should be emphasized to improve prognosis (28). Recently, Vockenhuber et al. reported a case of steroid-refractory fulminant ICI-associated myocarditis. The patient developed BVT and biventricular dysfunction following treatment with pembrolizumab (a PD-1 inhibitor) combined with chemotherapy. The patient improved after ruxolitinib was added to high-dose glucocorticoids (29). Combined with the two present cases, these literature findings suggest that BVT may be a significant ECG manifestation of ICI-associated myocarditis, suggestive of a complex clinical course and poorer prognosis.

In the current case series, the first case presented with acute myocarditis with preserved LVEF. After PD-1 inhibitor discontinuation and treatment with glucocorticoids and antiarrhythmic agents, the patient’s condition improved, and no further episodes of ventricular tachycardia occurred. In contrast, the second case developed fulminant myocarditis complicated with BVT within 3 weeks of the first sintilimab infusion, rapidly progressing to cardiogenic shock. Despite supportive therapies including ECMO, the patient ultimately died. ICIs have shown some benefit in refractory thymoma and thymic carcinoma, but with a higher risk of immune-related adverse events (irAEs) compared with other tumor types (30). This disparity in outcome is consistent with the broad clinical spectrum of ICI-associated myocarditis, ranging from mild forms of myocardial inflammation to fulminant disease, and associated with variable clinical outcomes (31). Consequently, promptly identifying ICI-associated myocarditis is critical. Both patients developed BVT during PD-1 inhibitor therapy, accompanied by acute chest symptoms and elevated troponin levels, which immediately alerted us to the possibility of myocarditis. CMR findings in the first case showed transmural myocardial enhancement in both ventricles, supporting the diagnosis of active myocarditis. The second case could not undergo advanced imaging studies or biopsy due to rapid disease progression, and her diagnosis was primarily based on clinical presentation and the history of ICI therapy. These circumstances demonstrate that invasive procedures and advanced imaging are often limited in critically ill patients, further highlighting the value of ECG and myocardial biomarker monitoring. For patients receiving immune checkpoint inhibitors, previous studies have recommended dynamic monitoring of electrocardiograms and myocardial injury markers during treatment to enable early detection of cardiotoxicity. However, the optimal monitoring frequency and intervals remain uncertain (11, 32).

Current guidelines and authoritative reviews generally agree that in ICI-associated myocarditis, BVT/wide QRS tachycardia is an electrophysiological manifestation of inflammatory cardiomyopathy, suggesting that this condition may be accompanied by widespread inflammation and hemodynamic risks, necessitating heightened vigilance from the clinical team (6). Suspected ICI-associated myocarditis requires immediate ICI suspension/discontinuation and urgent evaluation. Once ICI-associated myocarditis is diagnosed or highly suspected, glucocorticoids should be promptly initiated as first-line therapy, particularly if the patient’s condition is unstable. Second-line immunosuppressive drugs and mechanical circulatory support should also be considered when necessary (33, 34). For steroid-refractory ICI-associated myocarditis, current evidence is insufficient to support the routine use of early combination therapy with second−line immunosuppressants. Preliminary data suggest that abatacept may be beneficial in selected cases, with a case series from France showing reduced myotoxicity−related fatality; in contrast, evidence for mycophenolate mofetil remains lacking (35). In cases with an increased risk of hemodynamic collapse, the clinical team should preemptively establish a strategy for mechanical circulatory support and capitalize on the extremely narrow therapeutic window, thereby improving treatment success (22).

In conclusion, our two cases of ICI associated myocarditis complicated with BVT provide valuable supplementary data to the currently limited literature and highlight the critical importance of urgent diagnostic assessment for these rare but severe cardiovascular irAEs. Regular ECG evaluation and monitoring of myocardial biomarkers may enable proactive intervention during the reversible phase of ICI associated myocarditis, thereby potentially improving patient outcomes. Nonetheless, these observations require confirmation in future randomized controlled trials.

## Patient perspective

The patient (Case 1) acknowledges our efforts and agrees to continue follow-up treatment and to publish the case. And the patient’s family (Case 2) likewise expresses their appreciation and consent to publication.

## Data Availability

The original contributions presented in the study are included in the article/**Supplementary Material**. Further inquiries can be directed to the corresponding author.
