# Supplementary material for: Case Report: Two cases of PD-1 inhibitor–associated myocarditis with bidirectional ventricular tachycardia
Source: Front Immunol. 2026 May 25;17:1832047. doi: 10.3389/fimmu.2026.1832047 (PMC13243262; doi:10.3389/fimmu.2026.1832047)
Supplement: Supplementary file 1 [file SupplementaryFile1.pptx]

## Slide 1
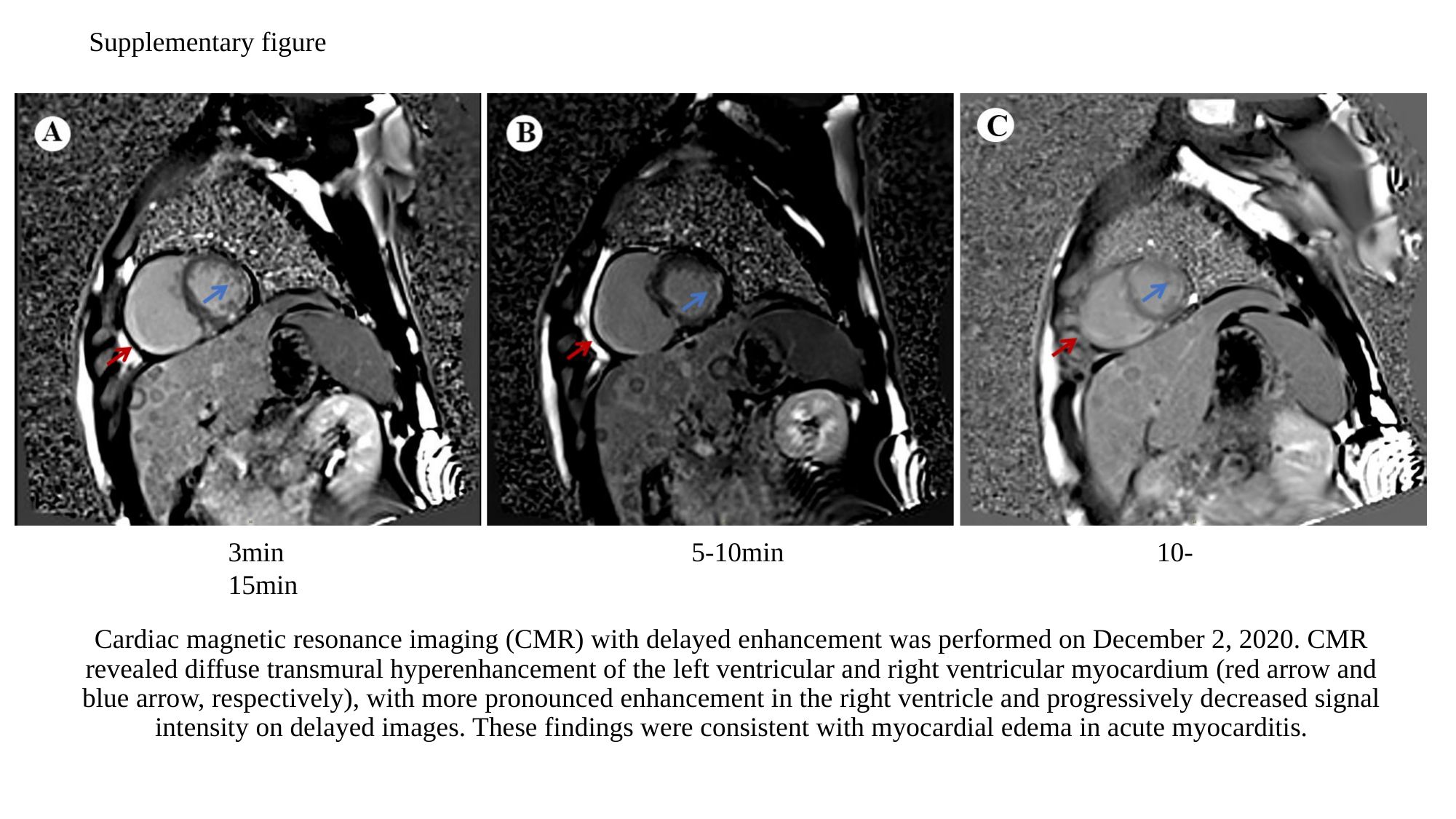

Supplementary figure
3min 5-10min 10-15min
Cardiac magnetic resonance imaging (CMR) with delayed enhancement was performed on December 2, 2020. CMR revealed diffuse transmural hyperenhancement of the left ventricular and right ventricular myocardium (red arrow and blue arrow, respectively), with more pronounced enhancement in the right ventricle and progressively decreased signal intensity on delayed images. These findings were consistent with myocardial edema in acute myocarditis.
